# Natural history of superficial head and neck squamous cell carcinoma under scheduled follow-up endoscopic observation with narrow band imaging: retrospective cohort study

**DOI:** 10.1186/s12885-016-2787-y

**Published:** 2016-09-21

**Authors:** Hiroshi Nakamura, Tomonori Yano, Satoshi Fujii, Tomohiro Kadota, Toshifumi Tomioka, Takeshi Shinozaki, Ryuichi Hayashi, Kazuhiro Kaneko

**Affiliations:** 1 Department of Gastroenterology, Endoscopy Division, National Cancer Center Hospital East, 6-5-1, Kashiwanoha, Kashiwa, Chiba 277-8577 Japan; 2 Pathology Division, Research Center for Innovative Oncology, National Cancer Center Hospital East, Kashiwa, Chiba Japan; 3 Department of Head and Neck Surgery, National Cancer Center Hospital East, Kashiwa, Chiba Japan

## Abstract

**Background:**

The incidence rate has been increasing for superficial head and neck squamous cell carcinoma (HNSCC) discovered through surveillance endoscopic study using narrow band imaging (NBI), a procedure mainly used for high-risk patients with esophageal squamous cell carcinoma (ESCC). However, there are few reports on the natural history of superficial HNSCC. The aim of this retrospective study was to investigate the natural history of superficial HNSCC.

**Methods:**

From January 2007 to December 2012, 535 consecutive histologically confirmed superficial HNSCCs at the oropharynx, hypopharynx, or larynx in 319 patients were detected by endoscopic surveillance examination by using NBI. Of those, 20 untreated and observed lesions fulfilled the eligibility criteria and were analyzed in this study.

**Results:**

Twenty lesions from 17 patients were analyzed. All patients were men ranging from 52 to 86 years of age, with a median age of 69 years. The median endoscopic follow-up period was 20 months (range, 6–71); 17 lesions progressed in size. In this study, four patients died; the causes of death were synchronous ESCC, synchronous HNSCC, acute myocardial infarction, and unknown causes. No patient died from progression of superficial HNSCC.

**Conclusions:**

Most superficial HNSCC has the potential to change progressively. Therefore, superficial HNSCC should be detected at an early stage and be treated less invasively, such as with endoscopic resection or partial resection.

## Background

Observation using a narrow band imaging (NBI) endoscope with magnified view makes it possible to visualize microvascular irregularities such as abnormalities of the intra-papillary capillary loop (IPCL). Several prospective randomized studies using this imaging technique have shown that the detection rate of superficial squamous cell carcinoma of the larynx and pharynx is enhanced [1–3].

Almost all superficial head and neck squamous cell carcinoma (HNSCC) can be cured with favorable prognosis by endoscopic resection (ER) or partial resection [4].

The synchronous or metachronous occurrence of esophageal squamous cell carcinoma (ESCC) and other HNSCCs is observed frequently; the former and latter rates are reported to be 14–83 % and 28 %, respectively [5, 6]. It is difficult to decide on a treatment course in such patients with synchronous or metachronous cancers. Generally, treatment for more advanced cancer takes precedence over that for other cancers when multiple synchronous cancers are detected at once. Therefore, treatment for superficial HNSCC is generally planned after concomitant advanced cancers have achieved a cure. Also, superficial HNSCC can be observed alone without any therapeutic intervention in cases where patients have an intolerable physical condition or concomitant further advanced stage of ESCC or HNSCC at other sites. However, there have been few reports regarding the natural history and prognosis of superficial HNSCC; therefore, therapeutic strategies are not well established. The aim of this retrospective study is to investigate the natural history of superficial HNSCC.

## Methods

### Patients

From January 2007 to December 2012, consecutive superficial HNSCCs were detected by oral endoscopic examination using NBI in high-risk patients who had prior or present HNSCC and ESCC. These lesions were located in the oropharynx, hypopharynx, and larynx, and were histologically confirmed. For these lesions, we analyzed the cohort that met the following eligibility criteria: 1) the lesion was the primary, 2) the lesion diameter was 20 mm or less, 3) the lesion was supposed to be clinically localized in the superficial mucosal region, 4) there was no lymph node or distant metastasis, 5) the initial treatment plan for HNSCC was observation, or planned ER if concomitant cancers, such as ESCC or HNSCC, had achieved a cure, 6) at least one follow-up endoscopic observation was performed after detection and the follow-up period was longer than 6 months, 7) there was no systemic chemotherapy for any cancer, and 8) there was no prior radiotherapy that involved the head and neck region.

Written informed consent was provided by the patients before all examinations and interventions. This is a retrospective study at a single institution, and the study protocol was approved by the institutional review board of the National Cancer Center on 23 March 2015 (approved clinical study number 2014–368) and was undertaken in conformity with the provisions of the Declaration of Helsinki. All information was collected from the database of our hospital or the patients’ medical charts and reports.

### Endoscopic examination

**Fig. 1:**
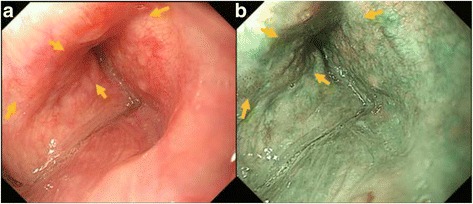
Endoscopic finding of superficial HNSCC in right piriform sinus. **a** White light imaging. Detection of lesion was difficult. **b** Narrow band imaging. Detection of lesion was easy as brownish area

Before endoscopic observation, 17.5–35 mg pethidine hydrochloride with or without 20 mg scopolamine butylbromide was administered intravenously to patients who did not have any contraindications to pethidine and scopolamine. For all patients, findings of NBI (GIF H260Z, GIF H260, GIF Q260; Olympus Medical Systems Co., Tokyo, Japan) were obtained at the initial surveillance or follow-up examination for HNSCC by oral esophago-gastric-duodenoscopic study (Fig. 1). The endoscopic images were retrospectively examined in detail with respect to location, macroscopic type, and size of each lesion. Lesion size was estimated by using forceps at a width of 6 mm when open (Radial Jaw [Boston Scientific, MA, USA]). The macroscopic type of lesion was classified according to the Japanese Classification of Esophageal Cancer (10th edition) [7]. Submucosal invasion was defined by endoscopic findings, such as enlargement of diameter, enhancement of thickness, change of protrusion and depression in the lesion, and the irregularity of the surface.

### Histological diagnosis

All the biopsy tissue specimens were fixed with formalin and embedded in paraffin to make a paraffin block. Thin 4 μm sections were cut from the blocks and stained with hematoxylin and eosin. Then, a histological diagnosis was performed by experienced pathologists by observing the sections under microscopic examination according to the World Health Organization classification of tumors (head and neck tumor, 2005). Superficial HNSCC in this study was defined as a lesion without invasion to the muscularis propria [8].

### Follow-up

Superficial HNSCC was followed-up with NBI endoscopy and physical examinations at 3–6 month intervals. In every endoscopic examination, we checked characteristics of lesions and measured depths of invasion and tumor diameters.

In our institution, superficial HNSCCs confirmed histologically are treated by ER or surgical resection in principle. Therefore, most superficial HNSCCs are treated when detected; however, some superficial HNSCCs are observed without any treatment when there are concomitant cancers or patients’ general conditions are poor. Moreover, some patients had dysphagia due to prior treatment for pharyngeal cancer. In these cases, superficial HNSCCs were observed without any treatment out of concern for worsening of patient swallowing ability. We continuously evaluated both the status of superficial HNSCC and that of concomitant cancers or the general condition of patients at each follow-up thereafter, and discussed the validity of treatment for superficial HNSCC. Furthermore, if a lesion infiltrated into the muscularis propria or deeper, or had lymph node and distal organ metastasis, we considered treating the lesion with more invasive therapy, such as surgical resection with lymph node resection or chemoradiotherapy (CRT).

Progression of lesion was defined by enlargement of tumor diameter and volume verified endoscopically. The lesions were divided into progression and non-progression groups, and then we compared patients’ age, lesion size, number of submucosal invasions, endoscopic follow-up period, and number of endoscopic examinations in each group. The endoscopic follow-up period was defined as the time between initial diagnosis and final endoscopic examination. The number of endoscopic examinations was counted as those that were performed between the initial diagnosis and the detection of enlargement.

### Statistics

The time to progression was measured from the date of diagnosis and the first date of enlargement of tumor diameter or volume verified endoscopically, and the tumor progression time curve was generated with the Kaplan-Meier method. This statistical analysis was done with SPSS 22.0 (IBM, Armonk, New York, USA).

## Results

### Characteristics of patients and lesions

**Fig. 2:**
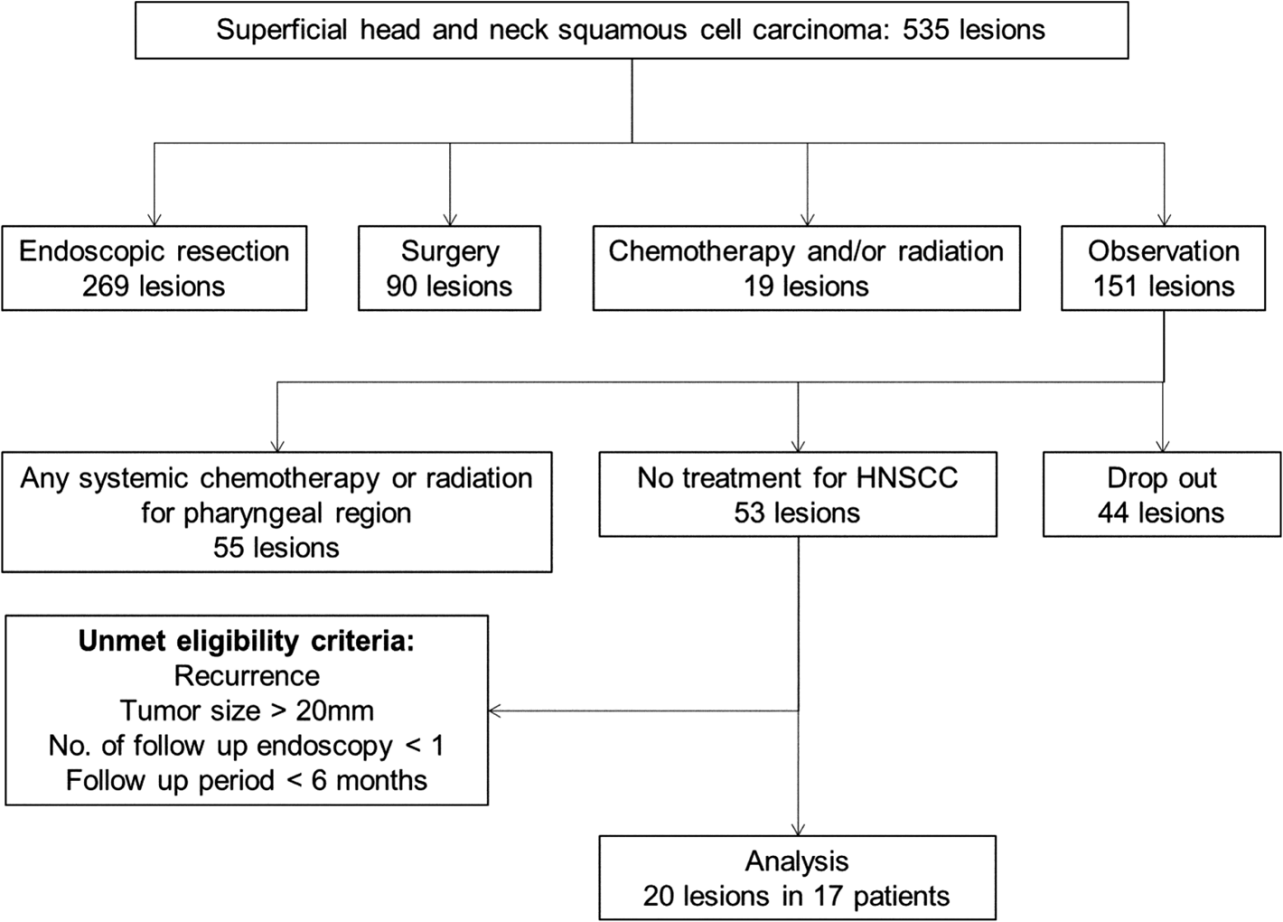
Patient enrollment in this study. Most lesions were treated by endoscopic resection, surgery, chemotherapy, and/or radiation. In the 151 untreated lesions observed, 20 lesions in 17 patients met the eligibility criteria. HNSCC, head and neck squamous cell carcinoma

Of the 535 consecutive lesions in 319 patients, 20 lesions (3.7 %) in 17 patients were enrolled and analyzed (Fig. 2). Of the 151 HNSCC lesions that underwent observation, 44 lesions did not receive follow-up endoscopy. The main reason for study drop out was due to the status of concomitant advanced cancer. Of the total lesions, 55 received systematic chemotherapy for cancer in other organs, or radiotherapy for another concomitant pharyngeal cancer. Therefore, we excluded these lesions due to the influence of treatment. The characteristics of patients and lesions are listed in Table 1. All 17 patients were men, and the median age was 69 years, ranging from 52 to 86 years. Of the 20 lesions, three were in the oropharynx, 12 were in the hypopharynx, and five were in the epiglottis. In regards to the macroscopic type of individual lesions, type 0-IIb was dominant. All lesions were endoscopically diagnosed as squamous cell carcinoma in situ. The median tumor size was 10 mm in diameter (range, 3–20 mm). Synchronous ESCCs, comprised of two early stage and five advanced stage, were present in seven patients. Synchronous HNSCCs, comprised of six superficial and one advanced stage at another site, were present in seven patients. Eight patients had prior ESCCs: three patients treated by ER, two patient treated by surgical resection, and three patients treated by CRT. In contrast, seven patients had prior HNSCCs: two patients treated by ER and five patients treated by surgical resection.

**Table 1:** Characteristics of superficial HNSCC (*n* = 17) and lesions (*n* = 20) in high-risk patients who had prior or present HNSCC and ESCC

Sex, *n* (%)	
Men	17 (100)
Women	0 (0)
Age, median (range), years	69 (52–86)
Synchronous cancer, *n* (%)	
HNSCC	7 (41)
ESCC	7 (41)
Prior cancer, *n* (%)	
HNSCC	7 (41)
ESCC	8 (47)
Lesion size, median (range), mm	10 (3–20)
Macroscopic type, *n* (%)	
Type 0-IIa	8 (40)
Type 0-IIb	10 (50)
Type 0-IIc	1 (5)
Type 0-IIa + 0-I	1 (5)
Location, *n* (%)	
Oropharynx	3 (15)
Hypopharynx	12 (60)
Larynx	5 (25)

*HNSCC* head and neck squamous cell carcinoma, *ESCC* esophageal squamous cell carcinoma

### Clinical course

**Fig. 3:**
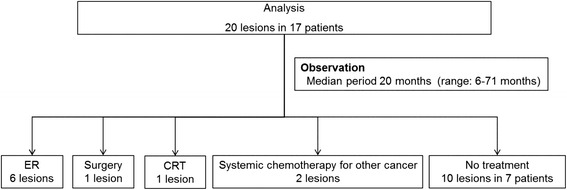
Clinical course of all lesions

**Fig. 4:**
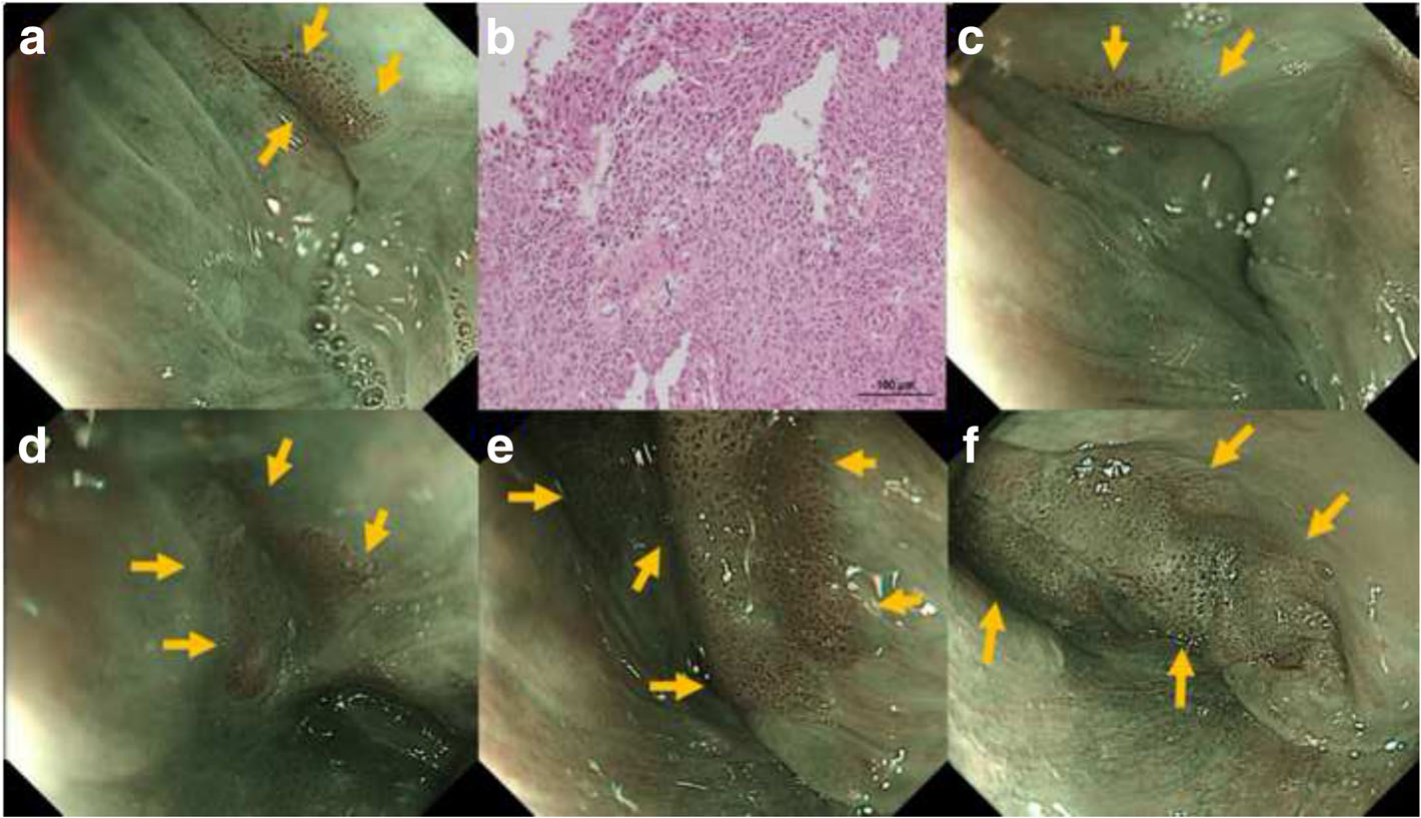
Endoscopic and pathological imaging of a progression lesion. **a** Brownish area with irregular IPCL in left pyriform sinus at diagnosis. The diameter was 5 mm. **b** The photomicrograph of the biopsy specimen shows the histopathology of squamous cell carcinoma in situ. (Hematoxylin and eosin staining × 20). **c** After 24 months. The lesion was not significantly different. **d** After 40 months. The lesion enlarged to 20 mm in diameter. **e** After 48 months. The lesion was larger and elevated. **f** After 58 months. An irregular surface and thickness of the lesion appeared. Submucosa invasion was suspected

The clinical course of all lesions is displayed in Fig. 3. The median endoscopic follow-up period was 20 months, ranging from 6 to 71 months (Table 2). Of the 20 lesions, 17 progressed during the follow-up period. Of the 20 lesions, eight lesions were treated. Six of the eight lesions were treated by ER after the median endoscopic follow-up period of 14 months (range: 8–42 months). Whereas, only one lesion that progressed after the 13 month observation period required treatment with surgical resection. That lesion was pathologically diagnosed with a surgical resected specimen as showing submucosal invasion. Another lesion was treated with CRT at 81 months after initial diagnosis. As shown in Fig. 4, the lesion was located in the hypopharynx, and its size was 5 mm. The endoscopic finding of progression and submucosal invasion appeared at 29 months and 58 months after commencement of follow-up, respectively. Of the eight treated patients, one patient died of synchronous ESCC at the conclusion of the study. Furthermore, two of the 20 lesions in two patients were treated with systemic chemotherapy, not for superficial HNSCC, but for advanced cancer in other organs at 22 and 24 months after initial diagnosis of superficial HNSCC.

**Table 2:** Endoscopic findings and clinical courses of the patients (*n* = 17) and lesions (*n* = 20) described in Table 1

Endoscopic follow-up period, median (range), month	20 (6–71)
Change of lesion size, *n* (%)	
Progression	17 (85)
Non-Progression	3 (15)
Treatment for HNSCC, *n* (%)	8 (40)
Endoscopic resection	6 (30)
Surgical resection	1 (5)
Chemoradiotherapy	1 (5)
Death, *n* (%)	5 (29)
Due to progression of superficial HNSCC	0 (0)

*HNSCC* head and neck squamous cell carcinoma

**Fig. 5:**
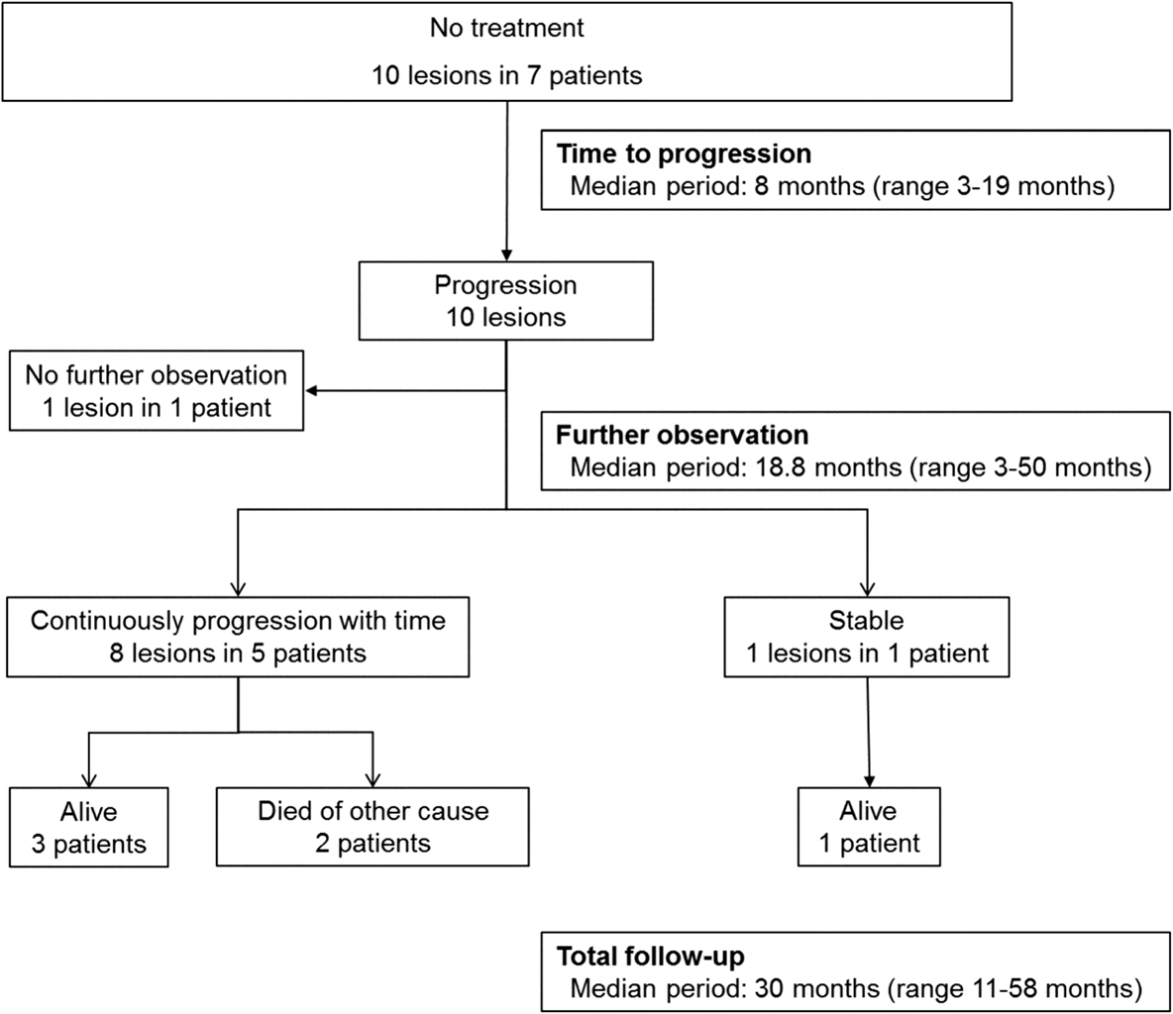
Clinical course of untreated lesions

The remaining 10 lesions in seven patients did not receive any treatment for superficial HNSCC. The clinical course of these lesions is displayed in Fig. 5. All lesions progressed after a median period of 30 months (range: 11–53). Seven of 10 lesions had endoscopic findings of submucosal invasion. Eight of nine lesions continuously progressed thereafter, and the other lesion followed up with endoscopy after progression was stable. In these patients, three patients died: one patient died of acute myocardial infarction, one patient died of synchronous ESCC, and one patient suddenly died of unknown causes. The other four patients remained alive, although no treatment had been performed.

**Fig. 6:**
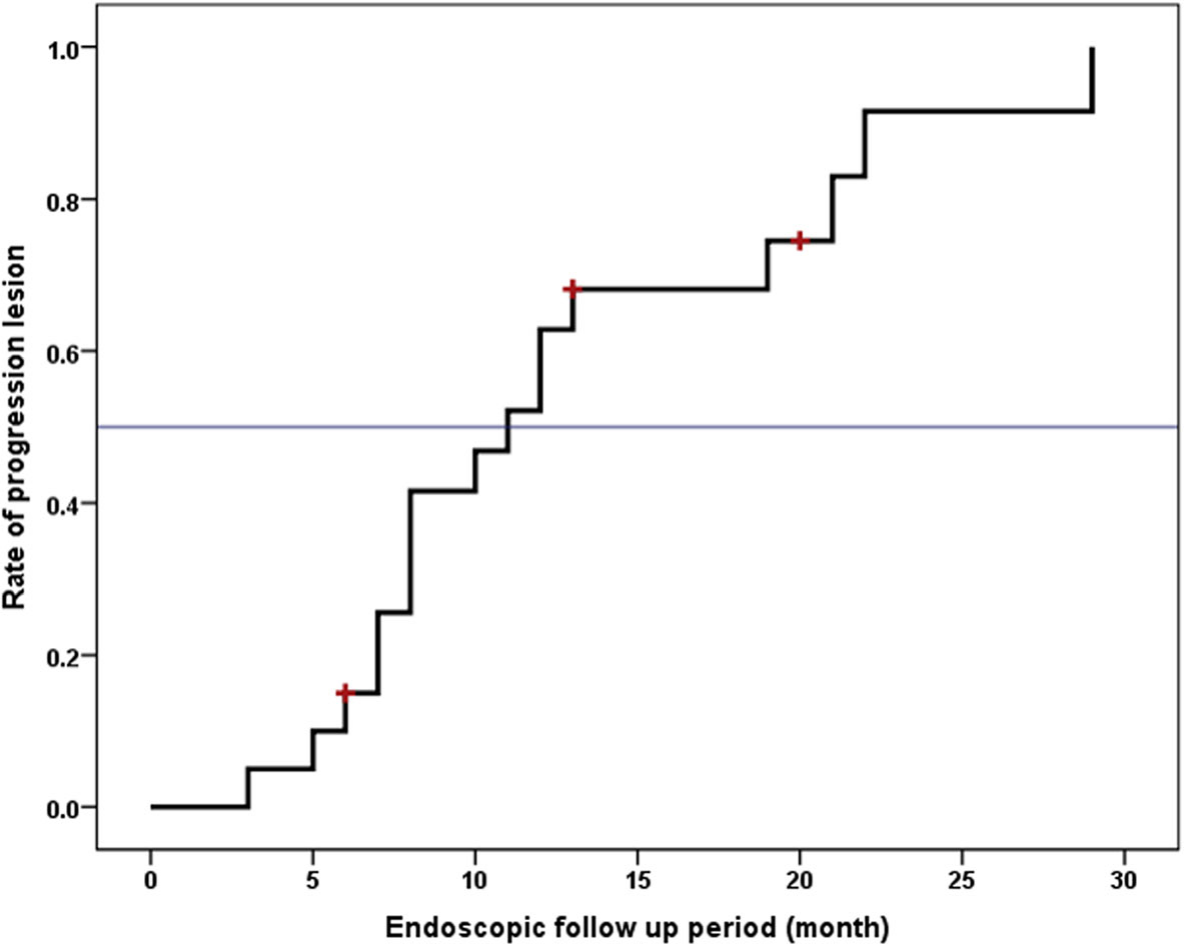
The Kaplan-Meier curve of tumor progression time in all lesions

The Kaplan-Meier curve of tumor progression time in all lesions is displayed in Fig. 6. All lesions progressed in 30 months of endoscopic follow-up and half of the lesions progressed in 11 months.

### Characteristics of progression and non-progression lesions

The characteristics of patients and those lesions with progression and non-progression are compared in Table 3. The median endoscopic follow-up periods of the progression and non-progression groups were 21 months (range, 6–71) and 13 months (range, 6–20), respectively (Table 4). In the progression group, the median period from diagnosis to appearance of lesion enlargement was 10 months (range, 3–29). The size of one lesion doubled at the earliest at 3 months after diagnosis. Endoscopic findings revealed submucosal invasion in nine lesions during follow-up and the median time to appearance was 21 months (range, 11–58).

**Table 3:** Characteristics in the two groups

	Progression	Non- Progression
	*n* = 17	*n* = 3
Age, median (range), years	69 (52–86)	70 (65–75)
Lesion size, median (range), mm	10 (3–20)	15 (8–15)
Macroscopic type, *n* (%)		
Type 0-IIa	6 (35)	2 (67)
Type 0-IIb	9 (53)	1 (33)
Type 0-IIc	1 (6)	0 (0)
Type 0-IIa + 0-I	1 (60)	0 (0)
Location, *n* (%)		
Oropharynx	3 (18)	0 (0)
Hypopharynx	9 (53)	3 (100)
Larynx	5 (29)	0 (0)

**Table 4:** Endoscopic findings between progression and non-progression groups

	Progression	Non- Progression
	*n* = 17	*n* = 3
Endoscopic follow-up period, median (range), months	21 (6–71)	13 (6–20)
Number of endoscopic examinations, median (range)	5 (1–10)	2 (2–5)
Time of tumor progression, median (range), months	10 (3–29)	
Number of submucosal invasion, (%)	9 (53)	
Time to submucosal invasion, median (range), months	21 (11–58)	

## Discussion

To our knowledge, this is the first study examining the natural history of superficial HNSCC. In this study, all lesions were diagnosed with NBI endoscopy and histologically confirmed with findings from biopsy specimens, and most of them progressed naturally. Takemura et al., reporting on the natural history of flat-type brownish lesions 5 mm or less in size in the oropharynx, found that the lesions did not change during 2 years of follow-up. However, pathological diagnosis was not performed and all lesions were described based on endoscopic findings alone [9]. Brownish areas of the pharynx vary from inflammation to invasive SCC. We previously reported the existence of basal cell hyperplasia (BCH), which is recognized as a small brownish area with NBI, but does not fulfill the pathological diagnostic criteria of neoplastic lesions such as SCC or dysplasia [8]. Most BCH in our previous report were 5 mm lesions or smaller. In contrast, most lesions enrolled in the present study were 5 mm or larger when detected. While most lesions were flat type, 85 % of them progressed. Therefore, the clinical course of superficial HNSCC confirmed histologically was different from those of flat-type brownish micro lesions. We believe that a 3 mm or larger superficial HNSCC is a significant lesion that requires careful follow-up or endoscopic intervention.

There are several reports about the depth of tumor invasion. It has been reported that macroscopic classification is related to tumor invasion. We previously reported that submucosal invasion was found in significantly more type 0-I and type 0-IIa lesions than in other macroscopic types. In addition to macroscopic classification, the rate of submucosal invasion increased significantly with larger tumor size [10]. Tateya et al. also reported that all lesions of type 0-I showed submucosal invasion, and 54 % of type 0-IIa lesions showed submucosal or muscular invasion. The ratio of submucosal or muscular invasion in each macroscopic type showed a significant difference [11]. Moreover, Fujii et al. reported there was significant correlation between the microvascular density of pharyngeal SCC and intra-epithelial SCC thickness and submucosal invasion [8]. As mentioned earlier, endoscopic findings can estimate tumor invasion and thickness. However, Taniguchi et al. reported that cervical lymph nodal metastasis of intra-epithelial SCC and submucosal invasive SCC of the pharynx were 0 % (0/77) and 9 % (7/75), respectively. Furthermore, in submucosal invasive SCC, tumor thickness of over 1000 μm was a significant risk factor for nodal metastasis and venous or lymphatic invasion [12]. As stated earlier, there were several reports about the endoscopic findings of submucosal invasive SCC. We decided the treatment plan of superficial HNSCC by those endoscopic findings. In our present study, although there were type 0-IIa lesions, no lesions had other obvious endoscopic findings indicating submucosal invasion at initial diagnosis. Considering these findings, it is suggested that all patients could be cured by ER. However, endoscopic findings, such as tumor enlargement or enhanced thickness, surface irregularity, and protrusions or depressions that suggested submucosal invasion appeared in nine lesions at the earliest at 11 months after diagnosis. Moreover, one progressed lesion treated with surgical resection at 13 months after diagnosis had invaded the submucosal layer, although there were no endoscopic findings during the initial and follow-up examination indicating submucosal invasion. These lesions might have a risk of nodal metastasis, and it might be difficult to cure only with ER or partial resection. This fact suggests that superficial HNSCC should be treated at an early stage, especially before tumor enlargement or appearance of endoscopic findings that suggest submucosal invasion. It is suggested that treatment for superficial HNSCC no larger than 20 mm commence within 1 year, because endoscopic findings indicating submucosal invasion appeared within 1 year after initial diagnosis in this study.

There are several limitations of this study. First, the median follow-up of 20 months might be insufficient to clarify the natural history of superficial HNSCC. Although 17 of the 20 lesions progressed in size during the endoscopic follow-up period, there were no patients who died from progression of superficial HNSCC. The follow-up period might be too short to reveal whether the superficial HNSCC would progress to be a cause of death. However, we believe that superficial HNSCC could be a cause of death because most lesions progressed to submucosal invasion in this study. Furthermore, we divided cohorts into two groups based on whether they progressed or not. We could not identify specific characteristics, such as endoscopic macroscopic type and location of lesions, in the progression group. Also, the follow-up period for the non-progression group was relatively short compared to that of the progression group, and two lesions were treated with ER and the other lesion was exposed to systemic chemotherapy in non-progression group.

Finally, the number of patients and lesions was small in this study. Further study with a large number of cases and a longer follow-up period at a multicenter setting will be required to clarify the natural history of these lesions and to clarify decision criteria for treatment of superficial HNSCC.

## Conclusions

This study showed that most superficial HNSCC progressed in size naturally, suggesting they should be treated with less invasive treatment such as ER or partial resection when they are small, if the patient’s situation allows. Whereas, if patients have high comorbidities such as active cancers or a physical intolerability to surgery, these lesions should be carefully followed-up under endoscopic observation.

## Abbreviations

BCH: Basal cell hyperplasia
CRT: Chemoradiotherapy
ER: Endoscopic resection
ESCC: Esophageal squamous cell carcinoma
HNSCC: Head and neck squamous cell carcinoma
IPCL: Intra-papillary capillary loop
NBI: Narrow band imaging
SCC: Squamous cell carcinoma
